# Clinical analysis of prolonged viral clearance time in patients with lymphoma combined with novel coronavirus infection

**DOI:** 10.3389/fmolb.2023.1240175

**Published:** 2023-10-04

**Authors:** Ying Li, Chao Wu, Liming Fei, Qin Xu, Xianru Shao, Bangjie Chen, Gengyun Sun

**Affiliations:** First Clinical Medical College (First Affiliated Hospital), Anhui Medical University, Hefei, China

**Keywords:** lymphoma, lung cancer, SARS-CoV-2, viral clearance time, immunity

## Abstract

**Objective:** To compare the period of viral clearance and its influencing factors after severe acute respiratory syndrome coronavirus (SARS-CoV-2) infection between patients with lymphoma and lung cancer.

**Methods:** We retrospectively collected the clinical data of patients with lymphoma and lung cancer (118 cases) diagnosed with SARS-CoV-2 infection and hospitalized in the First Affiliated Hospital of Anhui Medical University between 1 December 2022, and 15 March 2023. Finally, 87 patients with prolonged virus clearance times were included and divided into lymphoma (40 cases) and lung cancer (47 cases) groups. We used the Kaplan-Meier method to draw a negative turn curve. We performed a univariate analysis of the prolongation of virus clearance time and a Cox regression model for multivariate analysis.

**Results:** The median times for viral clearance in the lung cancer and lymphoma groups were 18 (95% confidence interval [CI] 15.112–20.888) and 32 (95%CI 27.429–36.571) days, respectively. Log-rank analysis showed a statistically significant difference (*p* = 0.048), and the lymphocyte count in the lymphoma group was lower than that in the lung cancer group (*p* = 0.044). We used the Cox regression model to conduct a multivariate analysis, which revealed that in lymphoma patients, the interval between the time of diagnosis and the time of SARS-CoV-2 infection <24 months (hazard ratio [HR]: 0.182, 95%CI: 0.062–0.535, *p* = 0.02), an interval between the last anti-CD20 monoclonal antibody treatment and the time of SARS-CoV-2 infection of <2 months (HR: 0.101, 95%CI: 0.029–0.358, *p* < 0.001), and a decrease in peripheral blood lymphocyte levels (HR: 0.380, 95%CI: 0.179–0.808, *p* = 0.012) were independent risk factors for prolonged viral clearance time.

**Conclusion:** Patients with lymphoma combined with SARS-CoV-2 infection had a longer virus clearance time than did patients with lung cancer. Moreover, the lymphocyte count in the lymphoma group was lower than that in the lung cancer group; therefore, the immune status of patients with lymphoma is lower than that of patients with lung cancer. An interval between lymphoma diagnosis and SARS-CoV-2 infection of <2 years, anti-CD20 monoclonal antibody treatment within the past 2 months, and a decrease in lymphocyte levels in the peripheral blood prolonged the virus clearance time in the patients in this study.

## 1 Introduction

In March 2020, the World Health Organization declared the disease caused by severe acute respiratory syndrome coronavirus (SARS-CoV-2) infection a global pandemic known as coronavirus disease 2019 (COVID-19) (Gopalaswamy and Subbian, 2021). Several risk factors for increased morbidity and mortality in COVID-19 patients have been identified, including male sex, hypertension, chronic lung disease, diabetes, immunodeficiency, and cancer. Therefore, identifying the potential risk factors for COVID-19 is of significant value for public health and healthcare policies (Jun et al., 2022). Patients with solid tumors or hematological malignancies often experience a more severe and rapid disease course requiring high-level intensive care. These patients are at higher risk of SARS-CoV-2 infection-related mortality than the general population. Lymphoma, a hematological malignancy characterized by severe immunosuppression, has been sparsely reported in combination with COVID-19; however, patients with lymphoma diagnosed with COVID-19 have a poor prognosis (Visco et al., 2022). In patients without lymphoma, the time to test negative for SARS-CoV-2 via reverse transcription-polymerase chain reaction (RT-PCR) is usually approximately 10 days (Li et al., 2021). However, patients with lymphoma are immunosuppressed and the virus may persist in the body for extended periods, affecting disease prognosis and survival outcomes. Notably, most individuals with COVID-19 exhibit mild to moderate infection but can rapidly progress from asymptomatic to acute respiratory distress syndrome, multiple organ dysfunction syndrome, and even death (Ballow and Haga, 2021). Although COVID-19 vaccination has effectively reduced the incidence of severe or critical illnesses, patients with lymphoma receiving the vaccine may not achieve effective protection because they often fail to generate sufficient antiviral immune responses (Kumar et al., 2017). Patients with lymphoma may have a higher risk of death owing to their inability to effectively clear the virus from their bodies. Therefore, assessing the continued presence of the novel coronavirus in these patients can assist in evaluating the risk of death. This study compared the duration of viral clearance after SARS-CoV-2 infection and its influencing factors between patients with lymphoma and those with lung cancer.

## 2 Materials and methods

### 2.1 Clinical information

We retrospectively collected the clinical data and performed follow-up of 49 patients with advanced lymphoma and 69 patients with advanced lung cancer who were diagnosed with SARS-CoV-2 infection and hospitalized at the First Affiliated Hospital of Anhui Medical University between 1 December 2022, and 15 March 2023.

### 2.2 Inclusion criteria, diagnostic criteria, and curative effect evaluation

We included patients who met the following criteria. **Inclusion criteria:** (Gopalaswamy and Subbian, 2021): confirmed prolonged viral clearance time (defined as a turnaround time >10 days); (Jun et al., 2022); confirmed lymphoma or lung cancer diagnosis through pathological, cytological, and relevant imaging examinations; (Visco et al., 2022); late-stage (III–IV) lymphoma or lung cancer in a stable disease (SD) state; (Li et al., 2021); diagnosed with SARS-CoV-2 infection; (Ballow and Haga, 2021); complete medical record documentation; and (Kumar et al., 2017) age≥18 years. This study included 87 patients (lymphoma group: 40 patients; lung cancer group: 47 patients. **Diagnostic criteria**: The diagnostic criteria for lymphoma staging followed the 2014 Lugano staging system, as follows: Stage I: involvement of a single lymph node area (I) or localized involvement of a single extranodal organ (I.E.,); Stage II: involvement of ≥2 lymph node areas on the same side of the diaphragm (II), possibly with limited involvement of localized extranodal organs in the same lymphatic drainage area (IIE); Stage III: involvement of lymph node areas above and below the diaphragm or involvement of the spleen in addition to above diaphragm involvement (IIIS); Stage IV: involvement of extranodal organs beyond the lymphatic drainage area (IV). Lung cancer was staged according to the 8th edition of the International Association for the Study of Lung Cancer (IASLC TNM) staging manual. **Curative effect evaluation:** The patients included in the study were categorized as having SD for the evaluation of therapeutic efficacy. Lymphoma response assessment was performed using the Lugano 2014 criteria: 1) complete response (CR), typically indicated by complete resolution on positron emission tomography-computed tomography (PET-CT) and computed tomography (CT) imaging, with the disappearance of lesions, normalization of organ size, absence of new lesions, and normal bone marrow morphology on CT. 2) Partial response (PR): fewer lesions, up to a maximum of six target lesions, and generally small existing lesions (often less than 5 mm × 5 mm), with some lesions gradually disappearing. Organ involvement in these patients is usually only minimally increased, and bone marrow remains unchanged compared to baseline. 3) Stable disease (SD): patients typically show no metabolic activity, with a five-point scale (5PS) score for target and extranodal lesions. Metabolic activity remains relatively unchanged compared to baseline. The bone marrow remains unchanged, the tumor size shrinks by <50% or increases by <25%, and no new lesions appear. 4) Disease progression: individual target lesions or extranodal lesions with a 5PS score of 4–5, tumor size increases by >25% or new lymphoma-related hypermetabolic lesions appear, and new or recurrent FDG avidity in the bone marrow. Lung cancer response assessment followed the Response Evaluation Criteria in Solid Tumors (RECIST) 1.1: CR, disappearance of all tumor lesions; PR, ≥30% decrease in the sum of the longest diameters of the target lesions; PD, ≥20% increase in the sum of the longest diameters of the target lesions, the appearance of new lesions, and/or definite progression of non-target lesions; and SD, lesion changes between PR and PD.

### 2.3 Study design

We performed a retrospective analysis by following up and comparing the clinical data of patients with advanced lymphoma and those with advanced lung cancer and defined positive results as viral infection status and negative results as viral clearance in patients via reverse transcription polymerase chain reaction (RT-PCR) testing. The time from positive to negative RT-PCR results in patients with lymphoma and lung cancer was recorded. The two groups of patients with prolonged conversion times were compared to investigate whether viral clearance time was longer in patients with lymphoma than in those with lung cancer. Multifactorial regression models have been used to investigate factors influencing prolonged viral clearance time in patients with lymphoma.

### 2.4 Study procedures, definitions, and follow-up

Follow-up was conducted by reviewing the outpatient and inpatient hospital information systems (HISs) and telephone follow-ups with a follow-up cutoff date of 1 April 2023. The collected data included demographic characteristics at enrollment, including sex, age, comorbidities (such as hypertension, diabetes, smoking, and history of hepatitis B), various laboratory test indices during hospitalization (such as peripheral blood leukocyte count, lymphocyte count, neutrophil count, albumin level, lactate dehydrogenase level, and C-reactive protein level), pathological staging and treatment of patients with lymphoma/lung cancer, the primary disease status at the time of SARS-CoV-2 infection (progression, remission, or stable), clinical symptoms (such as persistent fever ≥10 days and cough), the time interval between positive and negative RT-PCR test results in patients with lymphoma/lung cancer, the time interval between the diagnosis of the primary disease and SARS-CoV-2 infection in patients with lymphoma/lung cancer patients (more or less than 24 months) and the time interval between the last antitumor treatment and SARS-CoV-2 infection in such patients (more or less than 2 months).

### 2.5 Statistical treatment

Data management and statistical analyses were performed using IBM SPSS Statistics for Windows, version 27.0 (IBM Corp., Armonk, NY, USA). Measures that conformed to a normal distribution were expressed as x ± s, while those that did not were expressed as median (M) and upper and lower quartiles (Q1, Q3), using the Mann–Whitney U test. Count data were expressed as frequencies (percentages), and categorical variables in the study were tested using the X^2^ or Fisher’s exact tests. The Kaplan-Meier method was used to plot the transition curves. The log-rank test and Cox regression model were used for univariate and multivariate analyses, respectively. The hazard ratios (HRs) and the corresponding 95% confidence intervals (CIs) were calculated. Statistical significance was defined as *p* < 0.05.

## 3 Results

### 3.1 Analysis of novel coronavirus clearance times

**FIGURE 1:**
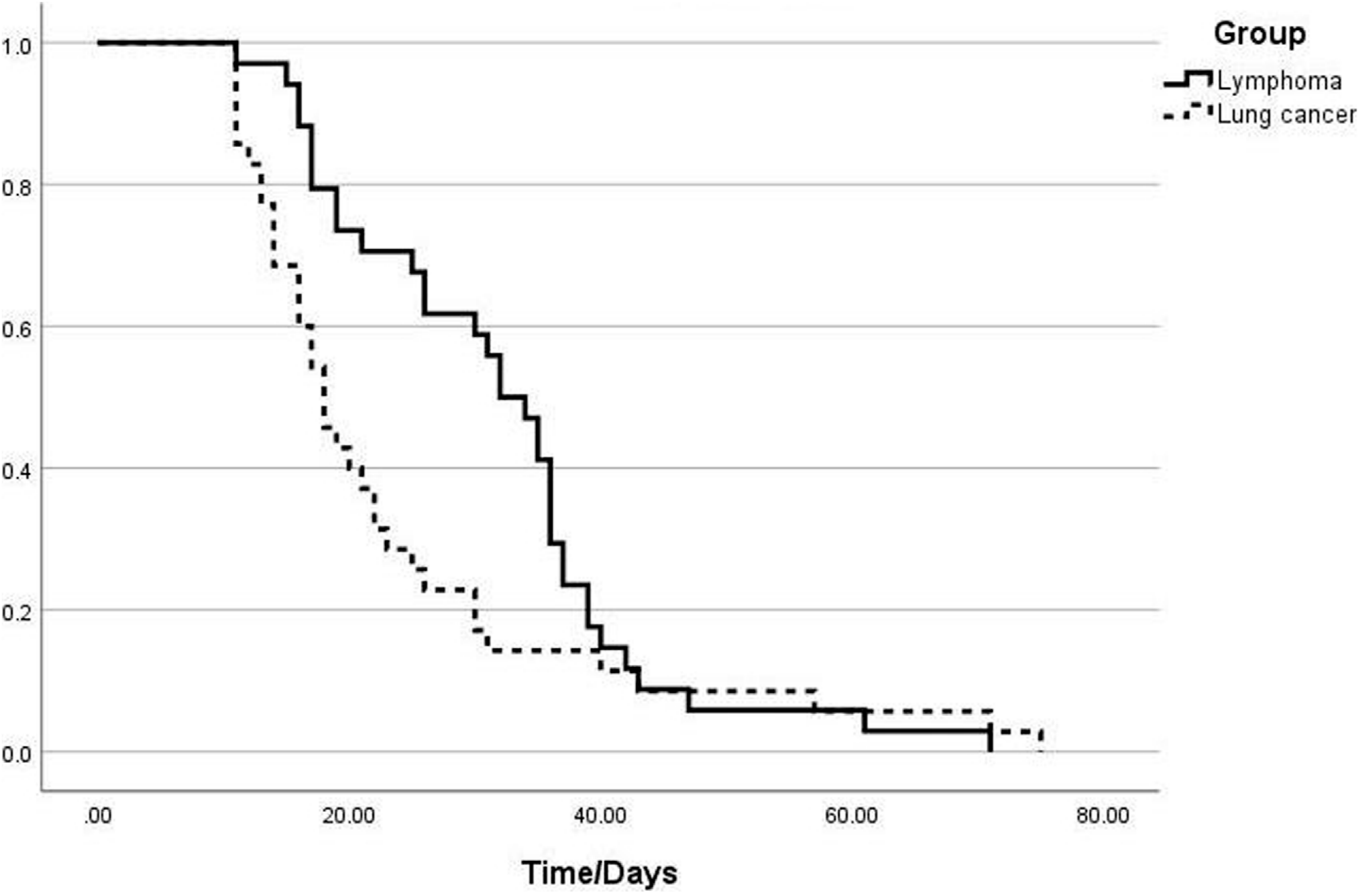
Analysis of the viral clearance time curves in the groups of patients with lymphoma and those with lung cancer.

The median times to viral clearance in the lung cancer and lymphoma groups were 18 days (95%CI 15.11–20.89) and 32 days (95%CI 27.43–36.57), respectively. Log-rank analysis showed a significant difference (*p* = 0.048). The Kaplan–Meier curve is shown in (Figure 1).

### 3.2 Univariate analysis of prolonged clearance time of the novel coronavirus

Univariate analysis showed lower peripheral blood indicators, including white blood cell, neutrophil, and lymphocyte counts, in patients with lymphoma than in those with lung cancer. In contrast, the lactate dehydrogenase levels were significantly higher in patients with lymphoma than in those with lung cancer. The lymphoma group had 18 patients with persistent fever (≥10 days), and the intergroup difference was significant (*p* < 0.05). In the lymphoma group, 25 patients received the anti-CD20 treatment regimen (Table 1).

**TABLE 1:** Univariate analysis of prolonged viral clearance time.

Indicators	Lymphoma group (*n* = 40)	Lung cancer group (*n* = 47)	*t/X* ^*2*^ */U* value	*p* Value
**Gender** [cases (%)]				
Male	26 (65.0)	40 (85.1)	0.101	0.751
Female	14 (35.0)	7 (14.9)		
**Age** [cases (%)]				
≥65 years	19 (47.5)	32 (68.1)	3.775	0.052
<65 years old	21 (52.5)	15 (31.9)		
**Underlying disease** [cases (%)]				
Hypertension	11 (27.5)	9 (19.1)	0.851	0.356
Diabetes	7 (17.5)	9 (19.1)	0.039	0.843
**Smoking history** [cases (%)]	9 (22.5)	15 (31.9)	0.959	0.327
**Laboratory indicators**				
WBC [×109/L, M (Q1, Q3)]	4.1 (2.4, 5.4)	6.14 (4.1, 7.9)	2.900	0.004
NEUT%[×109/L, s]	62.820.3	70.615.1	2.037	0.045
LYMPH%[×109/L, M(Q1, Q3)]	13.0 (7.9, 19.0)	20.5 (8.7, 27.0)	2.014	0.044
ALB[g/L, M(Q1, Q3)]	35.6 (32.1, 40.7)	35.9 (31.7, 41.5)	0.797	0.426
LDH[U/L, M(Q1, Q3)]	252.5 (189.0, 313.8)	206.0 (178.0, 269.0)	2.099	0.036
CRP[mg/L, M(Q1, Q3)]	23.6 (6.7, 48.1)	24.6 (5.9, 46.1)	0.130	0.896
**COVID-19 severity**				
Mild and moderate	37 (92.5)	40 (85.1)	0.548	0.459
Severe	3 (7.5)	7 (14.9)		
**Symptom**s [cases (%) (%)[[Example(%)[例](%)]				
Fever (≥10days)	20 (50)	12 (25.5)	5.564	0.018
Cough	19 (47.5)	16 (55.3)	0.232	0.675
**Treatmen**t[cases (%)]				
Anti-CD20	25 (62.5)	0	—	—
Chemotherapy	38 (95)	45 (95.7)	0.027	0.869

### 3.3 Multivariate analysis of prolonged clearance time of novel coronavirus

The results of multiple collinearity analyses of the statistically significant indicators of the univariate analysis suggested no collinearity among these indicators. These indicators were included in the multivariate Cox regression analysis. The results showed that the risk factors for prolonged viral clearance time were a time between lymphoma diagnosis and diagnosis of SARS-CoV-2 infection of <24 months (25 patients) (*p* = 0.002, HR: 0.182, 95%CI: 0.062–0.535); time between anti-CD20 monoclonal antibody treatment and diagnosis of SARS-CoV-2 infection of <2 months (18 patients) (*p* < 0.001, HR: 0.101, 95%CI: 0.029–0.358); and decreased peripheral blood lymphocyte levels in patients with lymphoma (*p* = 0.012, HR: 0.380, 95%CI: 0.179–0.808) were risk factors for prolonged viral clearance time (Table 2).

**TABLE 2:** Cox regression analysis of independent risk factors for prolongation of virus clearance time in patients with lymphoma.

Indicator	HR value	95% confidence interval (CI)	*p* Value
WBC	1.044	0.541∼2.012	0.898
NEUT%	1.930	0.954∼3.904	0.067
LYMPH%	0.380	0.179∼0.808	0.012
Diagnosis interval (<24 months)	0.182	0.062∼0.535	0.002
Anti-CD20 treatment interval (<2 months)	0.101	0.029∼0.358	<0.001
Aggressive tumor	1.121	0.897∼3.421	0.125
Fever (≥10 days)	1.754	0.944∼3.259	0.076

## 4 Discussion

The clinical manifestations of individuals infected with SARS-CoV-2 vary widely and range from mild flu-like symptoms to life-threatening respiratory failure. Acute respiratory distress syndrome (ARDS), caused by the release of proinflammatory mediators, an intense immune response, and endothelial damage is the leading cause of disease exacerbation or death (Batah and Fabro, 2021). Lymphoma is a group of non-solid tumors characterized by various immune dysfunctions, including congenital and acquired immune deficiencies, such as low serum immunoglobulin levels and impaired cellular and humoral immunity (Moyon et al., 2022), which place patients at risk of various infections, including that by SARS-CoV-2. Consistent with our findings, a previous study reported that a decreased lymphocyte count resulting from active antitumor treatments, such as anti-CD20 therapy, was a significant factor contributing to mortality in patients with lymphoma with concurrent SARS-CoV-2 infection (Lee et al., 2022). B and CD4^+^ T cells play essential roles in viral clearance; thus, patients with lymphoma patients, who have B and CD4^+^ T cell deficiencies, are unable to effectively and rapidly clear SARS-CoV-2, leading to a higher risk of disease exacerbation or death. Therefore, assessing the clearance of SARS-CoV-2 in patients can assist in evaluating patient prognosis. Hence, we conducted this retrospective analysis to study the prolongation of viral clearance time and its influencing factors to guide future disease treatment. For immunocompromised hosts, delayed viral clearance should be considered and the duration of antiviral treatment should be appropriately extended. Combination therapy with antiviral drugs can help achieve rapid viral clearance and control symptoms.

In our cohort, patients with lymphoma had a longer duration for SARS-CoV-2 clearance compared to those with lung cancer. The median times to seronegativity were 18 and 32 days in the lung cancer and lymphoma groups, respectively. Patients with lymphoma had lower levels of peripheral blood leukocytes (*p* = 0.004), neutrophils (*p* = 0.045), and lymphocytes (*p* = 0.044) than did those in patients with lung cancer, suggesting that patients with lymphoma have a reduced immune status compared to patients with lung cancer (Heo et al., 2019). The higher lactate dehydrogenase (LDH) levels in the lymphoma group could be related to the systemic characteristics of lymphoma. Most patients with lymphoma patients experience multiorgan and tissue damage, leading to elevated LDH levels in the peripheral blood. In contrast, patients with lung cancer typically exhibit increased LDH levels in pleural effusion (Wang et al., 2019). We defined persistent fever as fever symptoms lasting for >10 days. The proportion of patients with lymphoma with persistent fever was significantly higher than that of patients with lung cancer, with as many as half of the patients experiencing fever symptoms lasting for >10 days. However, high fever (>38.5°C) is rare, likely because of the continuous presence of the virus and stimulation of the immune response in the body. However, owing to the compromised immune system of patients with lymphoma, fever symptoms tended to persist compared to that in patients with lung cancer.

Decreased peripheral blood lymphocyte levels, an interval between lymphoma diagnosis and SARS-CoV-2 infection of <24 months, and an interval of anti-CD20 monoclonal antibody treatment of <2 months were high-risk factors for prolonged clearance time among patients with lymphoma with SARS-CoV-2 infection. The decrease in peripheral blood leukocyte and lymphocyte levels prevents patients with lymphoma from rapidly clearing the new coronavirus infection, similar to the general population, through CD4/CD8 and other mechanisms. As a result, patients show continuous viral carriage, leading to positive results on throat swab PCR nucleic acid testing.

In this study, we included a new indicator, namely, the time between the first definite diagnosis of lymphoma and the time of the first confirmed SARS-CoV-2 infection via nucleic acid testing. We classified this time interval according to a threshold of 24 months. Among the patients in this study, 15 and 25 patients with lymphoma showed intervals of ≥24 months and <24 months, respectively. The results of the multivariate regression analysis showed that an interval of <24 months between the diagnoses of lymphoma and COVID-19 was an independent risk factor for prolonged viral clearance. Generally, it is believed that the earlier the diagnosis of the underlying disease and the longer the course of the disease, the worse the patient’s physical condition, leading to a longer course of COVID-19 and an extended time to negative viral status. Interestingly, in the present study, the interval between lymphoma diagnosis and SARS-CoV-2 infection was negatively correlated with the viral clearance time. In other words, the earlier a patient was diagnosed with lymphoma, the shorter the viral clearance time after diagnosis with SARS-CoV-2 infection. One study also reported a linear decrease in the time for viral negativity via PCR with increasing time since lymphoma diagnosis. This may be because although patients have long-term lymphoma without disease progression or death, the disease is considered stable, and their immune status tends to be stable and closer to that of healthy individuals. Therefore, the viral clearance time was not prolonged.

We also included another research indicator, namely, the correlation between active antitumor chemotherapy within the past 2 months in patients with lymphoma and the viral clearance time. In this study, all 40 patients received lymphoma treatment, 25 of whom underwent treatment with the anti-CD20 regimen. We further divided these 25 patients into subgroups. Among them, 18 patients received anti-CD20 monoclonal antibody treatment within 2 months of confirmed SARS-CoV-2 infection, while seven patients had completed their last dose of anti-CD20 monoclonal antibody treatment >2 months previously. The results of the multivariate regression analysis revealed a negative between correlation anti-CD20 monoclonal antibody treatment >2 months before infection and the viral clearance time, demonstrating a prolonged clearance time in patients who had recently undergone chemotherapy. Rogado et al. (Rogado et al., 2020) reported that most patients with cancer have active disease or are undergoing aggressive treatment, which may result in higher mortality rates and may be related to cellular and humoral immune levels. Therefore, in clinical treatment, assessing the patient’s medical history and determining the time from the initial diagnosis and the most recent use of anti-CD20 monoclonal antibody treatment in patients infected with SARS-CoV-2 can guide the continuation or postponement of the next course of treatment. A risk-benefit assessment must be conducted for patients with cancer. If the benefits outweigh the risks, cancer treatment should continue (Linehan et al., 2022).

In clinical practice, aggressive tumors are generally associated with poor survival and prognosis. However, in this study, we confirmed no significant association between tumor invasiveness or noninvasiveness and the SARS-CoV-2 clearance time.

Our study has some limitations, including the small number of hospitalized patients with lymphoma with SARS-CoV-2 infection in our hospital. Additionally, some data were missing owing to the lack of CD+4/CD+8 examinations during the hospitalization of patients with lymphoma. Furthermore, because of the limited testing criteria at the time, patients with mild symptoms or asymptomatic individuals may not have been tested for COVID-19, and we only included patients who were active or planning to receive treatment. Moreover, while collecting data from the electronic medical records, we observed an increase in pulmonary fungal infections among some patients, which warrants further investigation in future studies. Future studies with larger numbers of patients and with additional data on CD+4/CD+8 levels and in patients with different levels of symptoms are also needed to verify our findings.

## Data Availability

The original contributions presented in the study are included in the article/Supplementary Material, further inquiries can be directed to the corresponding author.
